# Institutional Needs

**Published:** 1914-03-21

**Authors:** 


### INSTITUTIONAL NEEDS.

### A PROVINCIAL CENTRE FOR HOSPITAL FURNITURE.

Members of the medical profession, hospital committees, and all interested in hospital equipment, when visiting Liverpool, should certainly inspect the new showrooms of Messrs. White and Wright, 93 Renshaw Street, where a very large and varied assortment of aseptic hospital furniture is on view. The premises are so large as to form probably the most extensive hospital equipment centre in the provinces. The firm's goods are designed to stand the hardest wear and tear of the hospital ward. As Messrs. White and Wright are actual manufacturers, they are in a position to place these goods on the market at moderate prices, without the middleman's profit. Those who are interested in American hospital specialties will also find at Messrs. White and Wright's the latest patterns in operation tables, lockers, trolleys, etc. The firm are the local agents for Messrs. Kny, Scheerer and Co., of New York, whose importations come direct to Liverpool.

### "HERO" LIQUEUR WHISKY.

We have received a sample of Messrs. A. Dewar Rattray's "Hero" Old Scotch Liqueur Whisky, twenty years old, which well preserves the high standard of mildness and maturity, with pleasant flavour and aroma, which we have noted previously, of their products. The institutional cook who requires a whisky to flavour jellies and special diets may be cordially recommended to use it, and where alcohol is prescribed the physician may rely on a product free from adulteration if he specify the "Hero" brand. Where a whisky of this nature is obtainable at the reasonable prices demanded by Messrs. Rattray, whose address is 188 Dumbarton Road, Partick, Glasgow, those who have patients to prescribe or cater for cannot do better than place their orders with this firm, whose price list, including certain carefully selected wines, can be had on application.

### THE "EUREKA" BANDAGE.

On various occasions Mr. Vincent Wood's "Eureka" Crêpe Velpeau Bandage has been described and favourably noticed in these columns. Adjustable, light, and porous, while at the same time free from rubber, these bandages have a marked superiority for the treatment of varicose ulcers which is apparent to all who know the difficulties of procuring a bandage which possesses the required character without the disadvantages of the ordinary quality which is obtainable. The firm, whose offices are at Victoria House, Albion Place, Blackfriars Bridge, S.E., also manufacture abdominal binders in two widths, which possess not merely high quality but also the same reasonable cost which is associated with its productions, and the "Eureka" is a mark of genuine quality which has stood the test of time, and indicates a quality of article that it would be very difficult to rival. Institutional officers and medical men generally would be well advised to consult the price list of this firm, in which specifications may be studied with the knowledge that the peculiar qualities required for bandages of this class will be found in any orders that may be given.

### INVALID LIFTERS.

The recumbent invalid lifters manufactured by Mr. Arthur Skeffington, 49 Ulundi Road, Blackheath, S.E., have long been recognised as models of ingenuity. His latest catalogue contains not merely his accustomed devices but a new model, designed to give a bed-pan without moving the patient. The illustrative diagram, which should be consulted in this catalogue to appreciate the effectiveness and simplicity of the new model, shows how the sheet has to be folded and clamped, and then by turning the handles the manner in which the sheet is tightened, so that while the patient thus raised lies still the pan may be placed in position. After its withdrawal the sheet can be left either as first folded or it can be wound back to the roller at the foot. While personal inspection alone can reveal the simplicity of this ingenious device, a word must be found to mention the special lifter which Mr. Skeffington made for a patient with an injured spinal cord. The lifter was designed to enable him to wear an orthopædic corset which previously it had been a matter of great difficulty to get adjusted. By means of slings and a simple device one person was able to put on the corset, which it had required three or more attendants to put on, so difficult was the lifting of the patient. At the International Medical Exhibition last year a gold medal was awarded this firm, and all who realise the tremendous strain involved on both patients and nurses in cases where much lifting and raising are necessary will appreciate the scientific ingenuity which has been applied to these models, which are labour-saving in the most accurate sense.

### "REGULIN."

Much more detailed attention than formerly is given to the medical regulation of the bowels, and the bad old fashion of prescribing one or two stock drugs or mixtures to meet all conditions of obstruction has given way to the individual treatment of each by a method specially suited to it. For this reason "Regulin" has commanded attention; the main virtues claimed for it by the makers, the Regulin Syndicate, Ltd., 15 Cullum Street, E.C., that its action is mainly intestinal, that it is not a cathartic, are worthy of note. Sold in various forms: in a box of 1 oz. at 1s. 1½d., in tablets prepared with chocolate, a tube of which, containing twenty-five, is obtainable at the same price, and also in biscuits. "Regulin" is easily administered, tasteless, and carefully prepared. It may be commended to the notice of all who require a vegetable product as an aperient, and is prescribed with success in the conditions which it is specially designed to overcome.

### INSTITUTIONAL WASTE DISPOSAL.

The elimination of waste products is a characteristic of modern scientific and engineering developments, and the problem for institutions like hospitals and sanatoria is one which has received increasing attention of late years. Realising that both ordinary refuse and infectious materials have to be disposed of by institutions of this class, Messrs. Meldrum, whose works are at Timperley, near Manchester, and whose London office is at 92 Grosvenor Road, Westminster, have given much attention to high-temperature destructors, the smallest size of which can deal with up to 30 lb. an hour. This firm have also made a well-known feature of forced-draught furnaces and mechanical stokers, which are employed by numerous institutions. The destructors are constructed to avoid giving off offensive gases unburnt, and a special fire-brick hearth is provided for semi-liquid material, the fumes of which, while drying, are driven over the incandescent fire. The "Koker" and "Sprinkler" mechanical stokers, each of which can be worked with or without forced draught, are also established products of the firm, the institutions which have adopted Meldrum's patterns being so numerous as to form the best testimony to their value and efficiency. Detailed consideration is not possible here, but it may be noted that designs are willingly made for special requirements.

